# Do the Marital Statuses of Adult Offspring Affect Their Parent’s Mental Health? Empirical Evidence from China

**DOI:** 10.3390/ijerph191610133

**Published:** 2022-08-16

**Authors:** Yumiao Zhang, Wenbin Zang

**Affiliations:** School of Public Administration, Southwestern University of Finance and Economics, Chengdu 611130, China

**Keywords:** child-parent, marital status, mental health, China

## Abstract

Given the aging population, various issues pertaining to the elderly attract attention, including their mental health. Using data from the China Health and Retirement Longitudinal Survey (CHARLS), and adopting a propensity score matching (PSM) method, this study investigated the impact of offspring’s marital statuses on their elderly parents’ mental health. Parental depression was positively correlated with single and divorced/separated offspring aged 30 and above; this was not the case with widowed children. We then analyzed the heterogeneous influence of offspring’s marital statuses on parents’ mental health based on gender, region, and educational background, further expanding the research.

## 1. Introduction

With increasing life expectancy and declining fertility rates, population aging has become a worldwide problem [1], and issues related to older populations have attracted strong attention. Compared with other age groups, the elderly have the highest suicide risk rate [2]; therefore, the mental health problems of the elderly cannot be ignored. As they age and terminate their careers, the social circle of the elderly becomes gradually reduced [3,4], and children become the material and spiritual support for older parents. A great deal of academic research has explored the impact of progeny on the mental health of older adults. For example, scholars have examined the effects of child-parent contact, child-parent support, and child-parent residential distance on parental mental health [5,6,7,8].

In recent decades, people’s choices and behaviors regarding marriage have changed in both Western and Asian countries, as evidenced by a delay in the average age of first marriage, an increase in the proportion of singles and premarital cohabitation, a significant decline in fertility rates, and an increase in divorce rates [9,10,11,12,13,14,15,16,17,18]. Studies have shown that marital status is an important determinant of mental health [19,20] and that the differing marital statuses of offspring also have intergenerational effects on parental mental health [21,22,23]. However, the current academic research regarding the impact of children’s marital statuses on parents’ mental health is not sufficient. Milkie et al. [21] looked at children’s marital problems in their study on the impact of children’s negative life events on parents’ mental health, but this only included children’s divorce/separation processes. Chen and Tong [23] examined the effects of single children on parental mental health compared to married children. Arguably, there is no comprehensive literature examining the effects of the marital statuses of children (married, divorced/separated, widowed and single) on parental mental health, and in-depth analysis of whether the impact varies with parents’ characteristics.

In China, under the influence of Confucian culture, marriage is considered a social norm (men have the responsibility to father an heir, while women bear children); therefore, people in the past entered marriage early, and most marriages were decided on or arranged by their elders [24]. The older generation tends to pursue a stable married life, and until the 1980s, marriage was still the choice of most people. At that time, divorce, premarital cohabitation, and other non-traditional marriage practices were still very rare [25]. With the passing of time, however, the marital attitudes and behaviors of Chinese residents have changed, similar to those in other countries, such as the delay of the age of first marriage, the rise of divorce rates, and the gradual popularity of cohabitation [16,26]. The attitude of today’s young people towards marriage and their choices related to relationships and marriage have fundamentally disrupted the traditional concepts of their parents. How do children’s marital statuses affect their parents? Do the effects differ across parental characteristics? These are the questions driving the present research paper. The current study used data from the China Health and Retirement Longitudinal Survey (CHARLS), and adopted a propensity score matching (PSM) method to explore the effects of children’s marital statuses on parents’ mental health, further enriching and advancing research on the effects of children on parents’ mental health.

The remainder of this paper is organized as follows. A literature review is presented in Section 2. Section 3 outlines our data processing and research methods. Section 4 mainly includes our estimation results, heterogeneity analysis, and robustness test. Section 5 summarizes the conclusions of this paper. Finally, policy implications are put forward in Section 6.

## 2. Literature Review

Based on the life course theory, parents and children tend to influence each other due to their very close relationship, and this interaction continues throughout their lives [27]. A large number of studies have confirmed that the characteristics of parents affect children’s health, cognition, ability, and social development to a certain extent; this includes, but is not limited to, income, education, mental health, marriage, and parenting style [28,29,30,31]. The relationship between parents and children not only brings many positive emotional experiences to parents (such as children’s emotional and instrumental support for parents), but also brings many negative emotional experiences (such as quarrels and alienation between children and parents) [32].

The role of parents endows them with the duty of caring for and looking after children. Moreover, parental care continues even after their children reach adulthood [21]. Studies have shown that children diagnosed with a severe illness often bring great mental pain to their parents, and the risk of parents suffering from mental illness increases significantly [33,34,35]. Furthermore, many other problems that children face can also be troublesome for parents. Milkie et al. [21] find that children’s unemployment can increase parents’ depression. Greenfield and Marks [36] established a cumulative index of 10 problems that children may encounter, such as emotional problems, marital or partner problems, money problems, study and work problems, and their regression showed that children with more problems had parents who reported poorer mental health. According to the stress process theory, children’s misfortune often create role strains for parents, diminish their sense of mastery and self-esteem, lower self-concept, and lead to stress, which negatively affect their parents’ mental health [37,38].

Parental expectations are defined as parents’ beliefs about what they want their children to achieve, such as academic success [39]. Marriage is a normative marker of adulthood [40], and parents always place certain expectations on their children’s marriage. Taking China as an example, under the influence of Confucian culture, marriage is considered a social norm; therefore, people in the past entered marriage early [24]. The older generation tends to pursue a stable married life and expected their children to be like them. If the children’s marital behavior does not meet the parents’ expectations, conflicts between children and their parents are likely to occur, which may have a negative impact on the well-being of parents. Umberson [41] found that the marital statuses of children affected the quality of the relationship between children and parents. Both divorced and unmarried children reported more strained relationships with their mothers than married children. Kalmijn and De Graaf [22] found that the transition of children’s status from single to married has a positive impact on the mental health of parents.

Fingerman et al. [42] found that parents are fraternal, and one successful child cannot predict the well-being of parents. Only the total success of multiple children in the family can bring happiness to parents. Based on the above literature analysis, we argue that parents, out of love and care for their children and the expectation of a happy marriage of children, may experience negative emotions such as worry and self-blame when any child in the family has marital problems or keep unmarried at a marriageable age, thus affecting their well-being. This paper will adopt scientific methods to reveal the impact of children’s marital statuses on parents’ mental health.

## 3. Data, Variables, and Statistical Analyses

To achieve the study objectives, this paper uses married children as a benchmark to comparatively analyze the effects of children of other marital statuses (divorced/separated, widowed, and single) on parents’ mental health. The marital status of a child is not a random event, but is a process of the child’s self-selection. Parents’ early socioeconomic status tends to influence their children’s achievements and development during their formative years [29,30,43,44], indirectly affecting their children’s socioeconomic status as adults. Family background and children’s characteristics jointly determine the children’s opportunities to become married [45]. Moreover, the parents’ marital history and marriage quality can also affect their children’s marriage choices [46]. In order to reduce selection bias, this study adopted the propensity score matching method (PSM) developed by Rosenbaum and Rubin [47].

### 3.1. Data Process

The data in this article were derived from the fourth wave of the China Health and Retirement Longitudinal Survey (CHARLS). This is a large-scale survey project hosted by Peking University, which aims to collect a set of high-quality micro-data representing mainland Chinese residents aged 45 and older. The national baseline survey was conducted in 2011–2012, with waves 2, 3, and 4 carried out in 2013, 2015, and 2018 respectively. In order to ensure the representativeness of the sample, the CHARLS baseline survey covered 150 countries/districts and 450 villages/urban communities across the country, involving 17,708 individuals in 10,257 households.

Referring to Chen and Tong [23], this study restricted the age of children to 30 years or above. There were two reasons for doing so. First, due to the fact that age at first marriage in China is rising [16], the age of marriageability of children as perceived by parents is correspondingly increasing, and 30 happens to be the traditional Chinese “independent year”. Therefore, we argued that parents would not have significant concerns about their single children until they reach an older age. Second, in general, the proportion of children who are divorced/separated or widowed at a younger age is small. Moreover, referring to the study by Milkie et al. [21], we regarded divorce and separation as the same in terms of marital status. There are two reasons for this. First, the sample size of the separation group was too small to support the PSM method. Second, both divorce and separation in some sense imply a breakdown of the marital relationship.

The sample was processed as follows. First, we removed individuals with missing data (n = 4481). Second, we excluded individuals without children and individuals with at least one child under the age of 30 (n = 6322). Third, in order to ensure that there were individuals with corresponding variables in the control and treatment groups, we excluded a very small number of single individuals (n = 6). Finally, we excluded individuals whose children’s marital status corresponded to more than one status (widowhood, divorce/separation, and singlehood) to avoid the existence of multiple non-married states among the children of a single family (n = 107). The final sample size was 8900.

### 3.2. Empirical Model

We classified the sample into one control group and three treatment groups. The control group was the married group; all children in this group were married. Treatment group I was the single group, where at least one of the children was single and none were divorced/separated or widowed. Treatment group II was the divorced/separated group, where at least one of the children was divorced/separated and none were single or widowed. Treatment group III was the widowed group, where at least one of the children was widowed and none were single or divorced/separated.

We used the Logit model to estimate the propensity score and used four matching methods to make the estimation results more robust, namely, k-nearest neighbor matching (setting k to 4, also known as one-to-four matching, minimizes the mean square error [48]), caliper matching (caliper range = 0.01), kernel matching (using a quadratic kernel with a bandwidth of 0.06), and spline matching. Referring to Dehejia and Wahba [49] and Heckman et al. [50], we used the bootstrap method with 500 replications to correct standard errors. According to the present study’s research question, the average treatment effect on the treated (ATT) was applied for analysis:

$$ATT=E(Y_{1i}-Y_{0i}|D_{i}=1)=E(Y_{1i}|D_{i}=1)-E(Y_{0i}|D_{i}=1)$$

wherein, *D*_i_ = 1 indicates the treatment group, *E*(*Y*_1i_|*D*_i_ = 1) represents the average mental health of individuals in the treatment group, and *E*(Y_0i_|D_i_ = 1) denotes the average mental health of matched individuals in the control group.

### 3.3. Variables and Measurement

#### 3.3.1. Outcome Variables

In this study, parents’ mental health was described in terms of the level of depression they experienced. The CHARLS used the 10-item version of the Depression Scale of the Epidemiological Research Center (CESD-10; Andresen et al. [51]) to assess the respondents’ level of depression over the course of a week. Among the total 10, 8 questions were negative statements (such as “I feel depressed”) and the other 2 were positive (such as “I am full of hope for the future”). Each question had four response options: little or not at all (less than 1 day), not too much (1 to 2 days), sometimes or half of the time (3 to 4 days), and most of the time (5 to 7 days). Subsequently, 0−3 points were assigned to the four options (positive questions were assigned in the opposite direction). This created an index reflecting a respondent’s degree of depression after adding up the scores for all 10 questions. The higher the index score, the higher the respondent’s degree of depression.

#### 3.3.2. Treatment Variables

Three treatment variables were used: whether at least one of the children is single (Yes = 1, No = 0); whether at least one of the children is divorced/separated (Yes = 1, No = 0); and whether at least one of the children is widowed (Yes = 1, No = 0).

#### 3.3.3. Covariates

After referring to the relevant literature, we selected a series of variables that affect the mental health of parents or the marital statuses of their children. We controlled the parents’ age, gender, educational level, region, marital status, number of chronic diseases, disability, and monthly household consumption. We also controlled the number of living children, the number of deceased children, the proportion of children with poor health, the proportion of children with high school education or above, financial support, co-living, and nearby living. Detailed explanations of partial covariates are given in Table A1 in Appendix A.

### 3.4. Descriptive Statistics

Table 1 presents the descriptive statistics for the control and treatment groups. The number of individuals in the married, single, divorced/separated, and widowed groups were 7056, 782, 840, and 222, respectively. In these four groups, the average scores for parental depression were 8.378, 9.260, 10.217, and 10.099, respectively. The average scores for depression in the widowed, divorced/separated, and single groups were significantly higher than those in the married group. In each group, the number of fathers was slightly less than the number of mothers (the percentages of fathers were 47.8%, 48.2%, 45.7%, and 43.2%, respectively). Across all the groups, the proportion of parents with high school diplomas or above was low (less than 16%). In terms of region, the proportion of rural parents exceeded 50% in all groups, while the proportion of urban parents was 41.5%, 40.2%, 44.3%, and 30.6%, respectively.

## 4. Analysis and Results

### 4.1. Matching Quality Analysis

The premise of the propensity score matching method is that the propensity scores of the treatment and control groups must have a relatively wide common support domain. If the domain is too narrow, samples will be lost, resulting in unrepresentative remaining samples and failure of the PSM method. We observed a total of 12 matching schemes and found that only one individual from the treatment group in two matching schemes was outside the common support domain, and the propensity scores of all individuals in the rest of the matches were in the common support domain, satisfying the common support assumption condition well.

**Figure 1:**
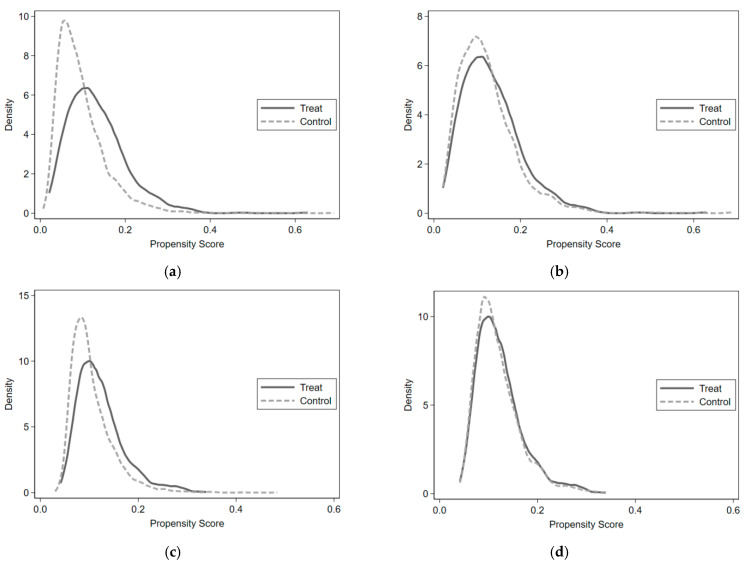

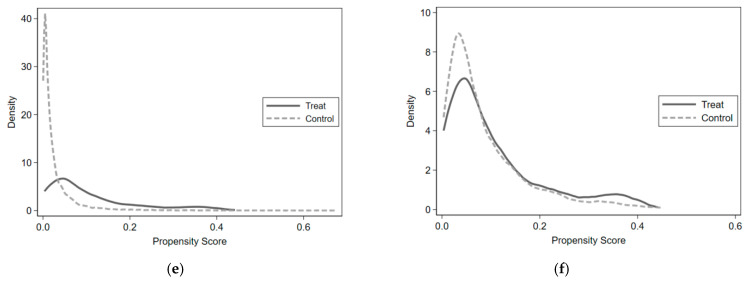
Kernel density functions of the treatment and control groups before matching and after k-nearest neighbor matching (k = 4). (**a**) Married-Single Group (Before Matching); (**b**) Married-Single Group (After K-nearest Neighbor Matching (k = 4)); (**c**) Married-Divorced/Separated Group (Before Matching); (**d**) Married-Divorced/Separated Group (After K-nearest Neighbor Matching (k = 4)); (**e**) Married-Widowed Group (Before Matching); (**f**) Married-Widowed Group (After K-nearest Neighbor Matching (k = 4)).

**Figure A1:**
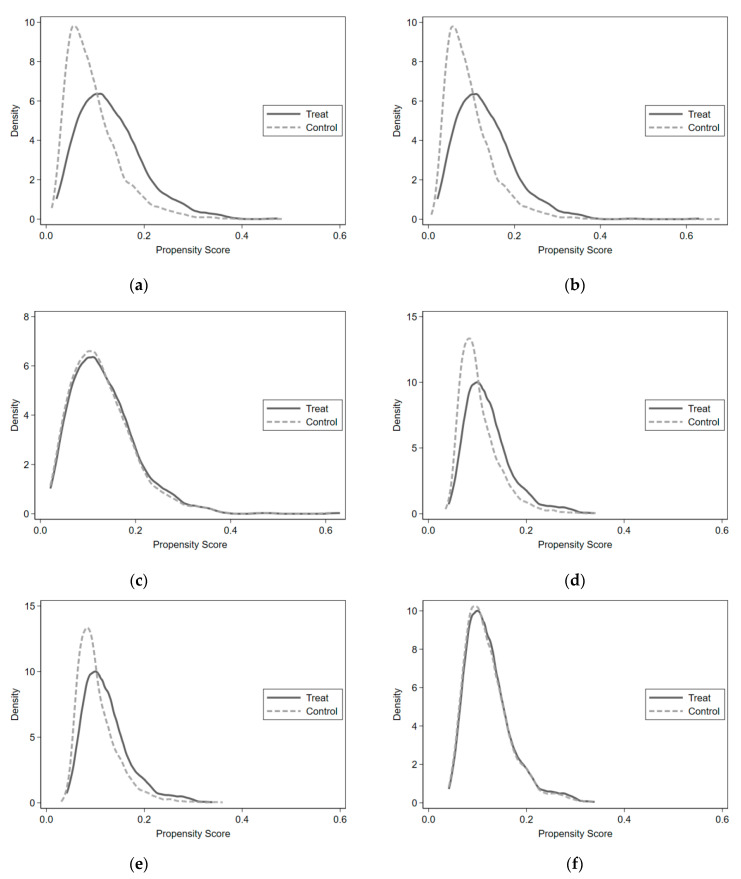

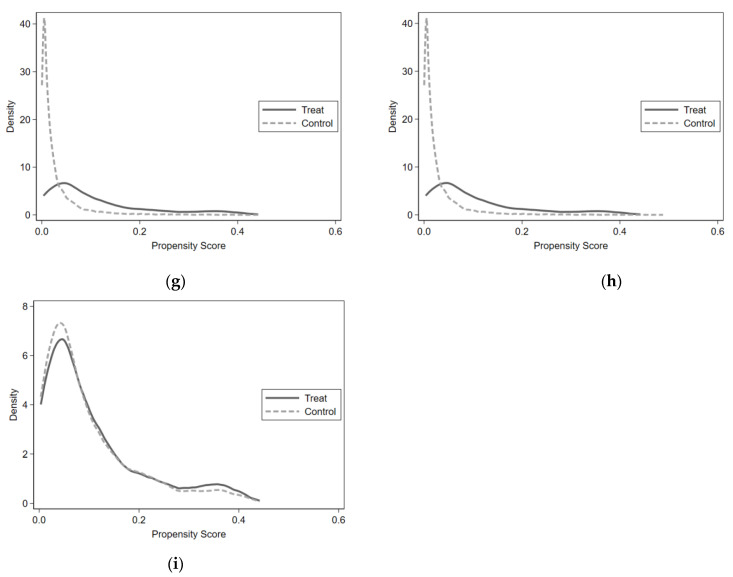
Kernel density functions of the treatment and control groups after caliper matching, kernel matching, and spline matching. (**a**) Married-Single Group (After Caliper Matching); (**b**) Married-Single Group (After Kernel Matching); (**c**) Married-Single Group (After Spline Matching); (**d**) Married-Divorced/Separated Group (After Caliper Matching); (**e**) Married-Divorced/Separated Group (After Kernel Matching); (**f**) Married-Divorced/Separated Group (After Spline Matching); (**g**) Married-Widowed Group (After Caliper Matching); (**h**) Married-Widowed Group (After Kernel Matching); (**i**) Married-Widowed Group (After Spline Matching).

In this study, we plotted the kernel density functions of the treatment and control groups before and after matching. As shown in Figure 1, the difference between the kernel density equation curves in the treatment and control groups before matching was large, and decreased after k-nearest neighbor matching (k = 4), and the trends converged, thus indicating good matching. The kernel density functions of the treatment and control groups after caliper, kernel, and spline matching are detailed in Figure A1 in Appendix A.

We further conducted balance tests for the four matching methods. The standardized percentage bias of almost all variables after four matching methods are less than 10%. Moreover, the *t*-tests of the differences in means of most variables after matching are not significant, indicating a good matching effect. To save space, the full results are detailed in Table A2 in Appendix A.

### 4.2. Matching Results Analysis

We used the Logit model to estimate the propensity scores, and the estimation results of the logit model for children’s marital statuses are detailed in Table A3 of Appendix A. The estimation results show that the following variables significantly affect the marital statuses of children in the household: parents’ age, marital statuses, the number of chronic diseases, monthly household consumption, region, the number of living children, the proportion of children with poor health, the proportion of children with high school education or above, co-living, nearby living, and financial support.

Table 2 shows the estimated average treatment effects on CESD-10 scores change in parents with four matching methods. As can be seen in Column A, compared to parents whose children were all married, the CESD-10 scores of parents with at least one child who was single increased by more than 0.8 points across the four matching methods. Furthermore, the four methods reached the 1% significance level. The results suggest an association between children’s singlehood and increased parental depression. Similarly, as can be seen in Column B, parental depression increased significantly by more than 1.2 points in all four matching methods when at least one child was divorced/separated. This provides evidence that increased levels of parental depression is associated with child divorce or separation. However, the results were different when the treatment group was the widowed group. Column C shows that parental depression increased significantly only in the kernel matching method, while it did not change significantly in the other three matching methods. Therefore, we can’t conclude that there is a significant association between the widowing of the offspring and increased depression in the parents. Why does the widowhood of offspring not increase parents’ depression? We have two explanations for this. One is that the death of a child’s spouse is mostly caused by objective factors, such as illness or accidental death [52], rather than the subjective choice of children, such as divorce/separation or remaining single. Therefore, parents have nothing to blame their children for. Another reason is that parents may not have strong negative emotions when facing the death of a child’s spouse due to illness, because they think their child is relieved and no longer dragged down by their sick spouse.

### 4.3. Heterogeneous Analysis

The influence of children’s marital statuses on parental mental health may vary with the characteristics of parents. Studies have found that women generally report worse mental health [53]. Copeland et al. [54] also found that women over the age of 65 have a higher prevalence of depression than men. Scholars have also typically considered parental gender differences when studying the intergenerational influence of children on their parents [5,21]. In China, there are large differences between urban and rural residents in terms of income levels, health care, etc. [55,56], and thus urban-rural differences are also commonly taken into account when scholars study Chinese issues [57,58]. In addition, because attitudes and beliefs about marriage can vary across educational levels [59], we believe that parents with different educational backgrounds will have different attitudes toward the marital status of their children. In this study, we also used the four matching methods mentioned in the previous section and corrected the standard errors with the bootstrap method (500 replications). Heterogeneity in the impact of children’s marital statuses on parents’ mental health was further explored from three perspectives: gender differences (male/female), urban-rural differences (rural/urban residents), and educational attainment differences (less than high school education, high school education or above). Due to the small number of individuals in the widowed group, very few remained in the treatment group following further grouping. Therefore, during this phase of the study, only the single group and the divorced/separated group were considered.

#### 4.3.1. Grouped by Male and Female

Table 3 shows the heterogeneous effects of parental gender. In Column A, the results indicate that compared with children who were married, children aged 30 or above who had never been married had a very different impact on the mental health of their father and mother. The degree of depression in mothers increased at least at the 5% significance level, but there was no significant change among fathers. This indicates mothers are more likely to experience negative emotions such as anxiety and worry when faced with their children’s singlehood. Whereas, in Column B, the results show that children aged 30 or above who are divorced or separated have a significant effect on both fathers and mothers. This indicates that both fathers and mothers show great concern for their children suffering from marital problems.

#### 4.3.2. Grouped by Urban and Rural Region

Table 4 shows the heterogeneous effects of the region. In Column A, our results indicate that compared with children who were married, children aged 30 or above who had never been married had a very different impact on the mental health of urban and rural parents. The level of depression among rural parents increased significantly (at the 1% level), but there was no significant change among urban parents, thus reflecting the differences in the attitudes toward marriage between urban and rural residents. Rural residents are more eager for their children to enter marriage, while urban residents hold a more inclusive attitude toward their single children. In Column B, the results indicate that compared with children who were married, children over 30 years who were divorced/separated had a significant impact on both urban and rural parents. This indicates that both parents, whether from urban or rural areas, display negative emotions toward their children’s divorce/separation.

#### 4.3.3. Grouped by Higher and Lower Parental Education

Table 5 shows the heterogeneous effects of education. As seen in Column A and Column B, single or divorced/separated children over the age of 30 had very different effects on the mental health of parents with different education levels. Parents who had less than a high school education were significantly negatively affected, while parents with a high school education or above were not. This indicates that parents with higher education are more accepting of the marital choices of their single or divorced/separated children.

### 4.4. Robustness Tests

In the first robustness test, we used multiple propensity score matching methods to estimate average treatment effects on parents’ CESD-10 score changes, which has been discussed previously. Next, we conducted robustness tests by varying the proxy variable for mental health and varying the model for estimating propensity scores.

#### 4.4.1. Measuring Mental Health with Life Satisfaction Index

Life satisfaction index is an important dimension for measuring mental health [60]. This study used life satisfaction as a proxy variable for mental health to re-examine the impact of children’s marital statuses on parents’ mental health. The CHARLS database provided us with a parental life satisfaction index. The relevant question had five options, scored from 1 to 5, from low to high, indicating a gradual decrease in satisfaction, which is similar to what the depression scale scores reflect. Four matching methods mentioned in the previous section were used here again, and the bootstrap method was also used to correct standard errors (500 replications). Table 6 shows the estimated average treatment effects on Life satisfaction changes in parents with four matching methods. We found that when at least one child was single or divorced/separated, parents’ life satisfaction was significantly lower than when all children were married; however, when at least one child was widowed, parents’ life satisfaction did not change significantly. This result is basically consistent with the previous conclusions regarding mental health measured by CESD-10 scores.

#### 4.4.2. Estimation of Propensity Score with Probit Model

In the previous section, we used the Logit model to estimate the propensity scores. In this section, we used the Probit model to estimate the propensity scores, again using the four matching methods mentioned in the previous section and correcting standard errors by the bootstrap method (500 replications). Table 7 shows the estimated average treatment effects on parents’ CESD-10 score changes with four matching methods (propensity score estimated by the Probit model). We found that, compared with parents whose children were all married, parents had significantly higher levels of depression when at least one child was either single or divorced/separated; furthermore, the level of parental depression increased significantly only in the kernel matching method, but did not change significantly in the other three matching methods when at least one child was widowed. This result is consistent with the previous findings, where propensity scores were estimated with the Logit model.

## 5. Conclusions

With the intensification of global aging, the health status of the elderly has attracted more and more attention. In this study, we comprehensively examined the impact of the marital statuses of children over 30 years on their parents’ mental health, utilizing CHALRS data and the PSM method. We found that the marital statuses of children were important determinants of parental mental health. Specifically, single children and divorced/separated children had a negative impact on parents’ mental health, while widowed children did not significantly affect their parents’ mental health. In terms of the heterogeneity analysis, we found that single children significantly affected the mental health of mothers, rural parents, and parents with less than high school education, while divorced/separated children significantly affected the mental health of parents with less than high school education. At last, this study has several limitations. First, our study did not make a mechanism analysis, i.e., we do not know how marital statuses of adult offspring influence their parents’ mental health. Second, due to the limitation of the research method of this paper, we did not further analyze whether the influence of offspring’s marital statuses on parents’ mental health varied with the gender of offspring. We will further expand the research on the above aspects in future studies.

## 6. Policy Implications

Based on our conclusions, this paper puts forward the following policy recommendations. First, in view of the current situation (in which the average age of first marriage and the divorce rate in China are both gradually increasing), it is imperative to impart education regarding marriage. For example, novel and interesting marriage education courses can be offered in middle school and university classrooms to encourage the younger generation to learn the responsibilities and obligations associated with married life. Various targeted marriage education lectures and activities can be held in communities, marriage registration offices, and other institutions to help cultivate the ability of couples to deal with various marital problems. Second, we ought to pay more attention to the mental health of middle-aged and older adults with single or divorced/separated children, especially mothers, rural parents, and parents with low educational levels. The families of middle-aged and older adults can be accessed and tracked at the community level and targeted mental health care services can be provided.

## Figures and Tables

**Table 1:** Descriptive statistics of the sample.

Variables	Married Group		Single Group		Divorced/ Separated Group		Widowed Group	
	Mean/%	SD	Mean/%	SD	Mean/%	SD	Mean/%	SD
Depression	8.378	6.497	9.260 ***	7.063	10.217 ***	7.056	10.099 ***	6.823
Age	66.029	7.475	63.739 ***	7.252	67.173 ***	7.060	74.212 ***	7.519
Gender	0.478	0.500	0.482	0.500	0.457	0.498	0.432	0.497
Education	0.123	0.329	0.155 *	0.362	0.113	0.317	0.018 ***	0.133
Married	0.852	0.355	0.849	0.358	0.802 ***	0.398	0.689 ***	0.464
Widowed	0.136	0.343	0.132	0.338	0.177 **	0.382	0.297 ***	0.458
Divorced/Separated	0.012	0.108	0.019	0.137	0.020 *	0.141	0.014	0.116
Disability	0.389	0.488	0.382	0.486	0.398	0.490	0.477 **	0.501
Number of Chronic Diseases	2.419	1.920	2.233 *	1.887	2.748 ***	2.044	2.514	1.999
Monthly Household Consumption	7.058	1.015	6.996	1.016	7.022	1.017	6.649 ***	1.143
Region	0.415	0.493	0.402	0.491	0.443	0.497	0.306 **	0.462
Number of Living Children	2.569	1.299	2.717 **	1.459	2.908 ***	1.334	4.248 ***	1.527
Number of Deceased Children	0.232	0.644	0.238	0.697	0.237	0.659	0.477 ***	0.921
Proportion of children with poor health	0.060	0.174	0.074 *	0.176	0.106 ***	0.220	0.118 ***	0.191
Proportion of children with high school education or above	0.365	0.416	0.431 ***	0.429	0.296 ***	0.377	0.134 ***	0.222
Co-Living	0.320	0.467	0.432 ***	0.496	0.392 ***	0.488	0.306	0.462
Nearby Living	0.221	0.415	0.162 ***	0.369	0.224	0.417	0.428 ***	0.496
Financial Support	0.834	0.372	0.753 ***	0.431	0.830	0.376	0.919 ***	0.274
N	7056		782		840		222	

Note: *, **, and *** indicate significant differences between the control and treatment groups at the 5%, 1%, and 1‰ levels, respectively.

**Table 2:** Estimated average treatment effects on CESD-10 scores change in parents.

Matching Method	A. Married-Single Group		B. Married-Divorced/ Separated Group		C. Married-Widowed Group	
	ATT	SE	ATT	SE	ATT	SE
K-nearest Neighbor Matching (k = 4)	0.848 **	0.327	1.247 ***	0.327	0.216	0.602
Caliper Matching	0.849 ***	0.241	1.289 ***	0.233	0.605	0.496
Kernel Matching	0.827 ***	0.238	1.478 ***	0.231	0.979 *	0.453
Spline Matching	0.823 ***	0.238	1.273 ***	0.232	0.635	0.446
N	7838		7896		7278	

Note: * *p* < 0.05, ** *p* < 0.01, *** *p* < 0.001.

**Table 3:** The heterogeneous effects of parental gender.

Matching Method	A. Married-Single Group				B. Married-Divorced/Separated Group			
	Male		Female		Male		Female	
	ATT	SE	ATT	SE	ATT	SE	ATT	SE
K-nearest Neighbor Matching (K = 4)	0.452	0.426	1.113 *	0.496	1.266 **	0.435	1.264 **	0.451
Caliper Matching	0.476	0.313	1.176 **	0.367	1.224 ***	0.327	1.314 ***	0.347
Kernel Matching	0.466	0.305	1.170 ***	0.354	1.325 ***	0.317	1.515 ***	0.336
Spline Matching	0.435	0.303	1.133 **	0.352	1.175 ***	0.319	1.296 ***	0.336
N	3752		4086		3759		4137	

Note: * *p* < 0.05, ** *p* < 0.01, *** *p* < 0.001.

**Table 4:** Heterogeneous effects of region.

Matching Method	A. Married-Single Group				B. Married-Divorced/Separated Group			
	Rural		Urban		Rural		Urban	
	ATT	SE	ATT	SE	ATT	SE	ATT	SE
K-nearest Neighbor Matching (K = 4)	1.278 **	0.433	0.226	0.467	1.207 *	0.476	1.538 **	0.473
Caliper Matching	1.268 ***	0.312	0.181	0.377	1.302 ***	0.350	1.259 ***	0.376
Kernel Matching	1.205 ***	0.304	0.293	0.367	1.541 ***	0.350	1.401 ***	0.367
Spline Matching	1.205 ***	0.306	0.313	0.367	1.264 ***	0.343	1.257 ***	0.361
N	4594		3244		4594		3302	

Note: * *p* < 0.05, ** *p* < 0.01, *** *p* < 0.001.

**Table 5:** Heterogeneous effects of parental education.

Matching Method	A. Married-Single Group				B. Married-Divorced/Separated Group			
	Lower Education		Higher Education		Lower Education		Higher Education	
	ATT	SE	ATT	SE	ATT	SE	ATT	SE
K-nearest Neighbor Matching (K = 4)	1.076 **	0.376	−0.151	0.659	1.178 ***	0.352	0.862	0.763
Caliper Matching	1.000 ***	0.282	0.043	0.555	1.359 ***	0.258	0.992	0.645
Kernel Matching	1.018 ***	0.279	−0.178	0.503	1.551 ***	0.255	0.983	0.584
Spline Matching	0.995 ***	0.280	−0.152	0.497	1.347 ***	0.254	0.940	0.582
N	6847		991		6931		965	

Note: ** *p* < 0.01, *** *p* < 0.001.

**Table 6:** Estimated average treatment effects on Life satisfaction changes in parents.

Matching Method	A. Married-Single Group		B. Married-Divorced/ Separated Group		C. Married-Widowed Group	
	ATT	SE	ATT	SE	ATT	SE
K-nearest Neighbor Matching (k = 4)	0.160 ***	0.039	0.154 ***	0.039	0.100	0.076
Caliper Matching	0.167 ***	0.030	0.159 ***	0.030	0.059	0.060
Kernel Matching	0.170 ***	0.030	0.165 ***	0.029	0.037	0.056
Spline Matching	0.168 ***	0.030	0.158 ***	0.029	0.036	0.056
N	7838		7896		7278	

Note: *** *p* < 0.001.

**Table 7:** Estimated average treatment effects on CESD-10 score changes in parents. (estimation of propensity score with Probit model).

Matching Method	A. Married-Single Group		B. Married-Divorced/ Separated Group		C. Married-Widowed Group	
	ATT	SE	ATT	SE	ATT	SE
K-nearest Neighbor Matching (k = 4)	0.699 *	0.322	1.367 ***	0.314	0.630	0.634
Caliper Matching	0.819 ***	0.242	1.283 ***	0.233	0.568	0.481
Kernel Matching	0.834 ***	0.239	1.471 ***	0.231	0.911 *	0.447
Spline Matching	0.827 ***	0.239	1.270 ***	0.232	0.595	0.446
N	7838		7896		7278	

Note: * *p* < 0.05, *** *p* < 0.001.

## Data Availability

The datasets used and analyzed during the current study are available from the corresponding author on request.

## Appendix A

**Table A1:** Explanation of the Partial Covariates.

Covariates	Explanation
Gender	Male = 1, Female = 0
Educational level	High school education or above = 1, Less than high school education = 0
Marital status	Married, widowed, or divorced/separated
Disability	Five questions were included in the questionnaire to obtain information about whether the respondents had physical disabilities, brain damage/intellectual disability, vision problems, hearing problems, or speech impediments. We regarded any respondents with one or more of the above conditions as having a disability.
Monthly household consumption	The variable was winsorized at 1% and 99% percentile, followed by taking the natural logarithm.
Region	Urban = 1, rural = 0.
Proportion of children with poor health	The number of children with poor health/the number of living children. The health status of children was obtained through the questionnaire: “How is the health of the child?” The options were “very good,” “good,” “average,” “bad,” and “very bad.” We considered the responses of “bad” and “very bad” as children with poor health status.
Proportion of children with high school education or above	The number of children with high school education or above/the number of living children.
Financial support	At least one child provides financial support for their parents = 1, otherwise = 0.
Co-living	At least one child living near parents = 1, otherwise = 0.
Nearby living	At least one child lives with their parents = 1, otherwise = 0.

**Table A2:** The results of the balance tests.

Variables	Before/After Matching	Married-Single Group			Married-Divorced/ Separated Group			Married-Widowed Group		
		Bias (%)	t	*p* > t	Bias (%)	t	*p* > t	Bias (%)	t	*p* > t
Age	Before	−31.1	−8.15	0.000	15.7	4.22	0.000	109.2	16.06	0.000
	After One-to-Four Matching	3.2	0.65	0.516	−2.3	−0.46	0.648	−0.5	−0.05	0.960
	After Caliper Matching	2.1	0.43	0.669	−0.6	−0.12	0.906	5.8	0.62	0.536
	After Kernel Matching	−3.5	−0.69	0.489	5.3	1.08	0.282	30.2	2.95	0.003
	After Spline Matching	2.8	0.57	0.568	−3.1	−0.61	0.541	−1.3	−0.14	0.886
Gender	Before	0.8	0.20	0.841	−4.2	−1.16	0.245	−9.2	−1.35	0.178
	After One-to-Four Matching	−0.5	−0.11	0.915	2.0	0.41	0.683	−1.2	−0.12	0.901
	After Caliper Matching	−0.1	−0.01	0.989	−0.3	−0.06	0.949	−0.1	−0.01	0.989
	After Kernel Matching	0.2	0.03	0.972	−1.7	−0.35	0.723	−1.5	−0.16	0.876
	After Spline Matching	−1.5	−0.30	0.762	−1.7	−0.34	0.732	1.8	0.19	0.848
Education	Before	9.1	2.51	0.012	−3.2	−0.85	0.393	−42.0	−4.76	0.000
	After One-to-Four Matching	0.2	0.03	0.972	1.8	0.38	0.706	0.9	0.18	0.854
	After Caliper Matching	−1.2	−0.22	0.824	0.2	0.04	0.967	−1.3	−0.25	0.804
	After Kernel Matching	1.3	0.24	0.811	−1.4	−0.29	0.768	−14.5	−2.06	0.040
	After Spline Matching	4.4	0.86	0.392	5.5	1.20	0.231	3.6	0.82	0.412
Married	Before	−0.8	−0.22	0.827	−13.2	−3.78	0.000	−39.4	−6.66	0.000
	After One-to-Four Matching	−3.4	−0.69	0.491	0.6	0.12	0.903	4.3	0.40	0.692
	After Caliper Matching	−0.6	−0.12	0.901	−0.5	−0.10	0.917	−1.9	−0.17	0.861
	After Kernel Matching	−0.7	−0.15	0.883	−5.4	−1.07	0.286	−9.2	−0.88	0.378
	After Spline Matching	−6.5	−1.31	0.189	−2.2	−0.43	0.666	−1.1	−0.10	0.918
Widowed	Before	−1.3	−0.35	0.728	11.3	3.25	0.001	39.8	6.81	0.000
	After One-to-Four Matching	1.0	0.20	0.845	−1.6	−0.32	0.751	−5.2	−0.48	0.633
	After Caliper Matching	0.6	0.11	0.911	0.2	0.04	0.966	2.2	0.21	0.837
	After Kernel Matching	0.0	0.01	0.996	4.4	0.87	0.384	9.3	0.88	0.379
	After Spline Matching	3.4	0.69	0.493	2.6	0.52	0.606	1.1	0.10	0.917
Divorced/ Separated	Before	6.0	1.77	0.077	6.8	2.08	0.038	1.6	0.24	0.812
	After One-to-Four Matching	7.3	1.47	0.142	2.8	0.54	0.587	3.0	0.33	0.743
	After Caliper Matching	0.3	0.05	0.962	1.0	0.18	0.857	−1.1	−0.11	0.910
	After Kernel Matching	2.1	0.38	0.702	3.5	0.67	0.502	0.5	0.06	0.955
	After Spline Matching	9.3	1.98	0.048	−0.9	−0.17	0.864	0.0	0.00	1.000
Disability	Before	−1.3	−0.35	0.728	1.8	0.50	0.618	18.0	2.67	0.008
	After One-to-Four Matching	1.2	0.23	0.815	2.5	0.51	0.609	−2.0	−0.21	0.835
	After Caliper Matching	1.3	0.26	0.797	0.6	0.12	0.901	−0.4	−0.04	0.968
	After Kernel Matching	0.9	0.18	0.859	1.3	0.26	0.795	3.7	0.39	0.700
	After Spline Matching	−2.1	−0.41	0.678	−5.8	−1.19	0.234	−5.5	−0.57	0.570
Number of Chronic Diseases	Before	−9.8	−2.57	0.010	16.6	4.66	0.000	4.8	0.73	0.468
	After One-to-Four Matching	4.2	0.87	0.385	0.0	0.01	0.995	−2.2	−0.23	0.822
	After Caliper Matching	0.4	0.08	0.940	0.3	0.07	0.948	−0.7	−0.07	0.944
	After Kernel Matching	−1.0	−0.20	0.841	6.0	1.21	0.225	1.9	0.20	0.839
	After Spline Matching	5.2	1.08	0.278	1.7	0.35	0.727	−2.5	−0.26	0.793
Monthly Household Consumption	Before	−6.1	−1.61	0.107	−3.5	−0.97	0.332	−37.8	−5.88	0.000
	After One-to-Four Matching	−0.3	−0.06	0.948	−4.2	−0.86	0.391	0.5	0.05	0.957
	After Caliper Matching	−1.0	−0.19	0.848	0.5	0.10	0.921	−1.4	−0.15	0.883
	After Kernel Matching	−1.5	−0.28	0.777	−0.9	−0.19	0.850	−11.1	−1.13	0.259
	After Spline Matching	−1.8	−0.34	0.732	−1.9	−0.40	0.690	−14.2	−1.48	0.139
Region	Before	−2.8	−0.74	0.460	5.6	1.53	0.125	−22.8	−3.25	0.001
	After One-to-Four Matching	4.1	0.81	0.419	0.5	0.10	0.920	4.2	0.47	0.641
	After Caliper Matching	0.0	0.01	0.994	0.4	0.09	0.930	−0.5	−0.06	0.954
	After Kernel Matching	−0.2	−0.04	0.970	2.2	0.44	0.660	−7.7	−0.83	0.406
	After Spline Matching	2.9	0.57	0.570	−0.2	−0.05	0.961	6.6	0.73	0.465
Number of Living Children	Before	10.8	3.00	0.003	25.8	7.14	0.000	118.4	18.86	0.000
	After One-to-Four Matching	3.8	0.69	0.488	−0.4	−0.08	0.933	5.5	0.50	0.614
	After Caliper Matching	5.4	1.02	0.307	0.6	0.12	0.902	3.6	0.31	0.754
	After Kernel Matching	5.6	1.06	0.288	9.9	1.96	0.051	31.6	2.73	0.007
	After Spline Matching	4.3	0.78	0.434	−0.5	−0.10	0.916	1.9	0.17	0.862
Number of Deceased Children	Before	0.9	0.25	0.802	0.8	0.22	0.826	30.9	5.51	0.000
	After One-to-Four Matching	−1.6	−0.29	0.770	1.4	0.28	0.777	−7.2	−0.62	0.536
	After Caliper Matching	1.4	0.27	0.788	0.0	0.01	0.996	2.2	0.20	0.842
	After Kernel Matching	1.3	0.24	0.807	0.4	0.09	0.927	9.5	0.89	0.374
	After Spline Matching	−3.4	−0.66	0.512	2.4	0.50	0.620	−1.7	−0.16	0.877
Number of Children with Poor Health	Before	8.1	2.15	0.032	23.0	6.98	0.000	31.6	4.86	0.000
	After One-to-Four Matching	−1.1	−0.20	0.839	4.9	0.91	0.363	−6.1	−0.56	0.574
	After Caliper Matching	−0.8	−0.15	0.880	3.4	0.64	0.526	−3.1	−0.29	0.773
	After Kernel Matching	1.4	0.25	0.799	11.1	2.13	0.034	5.1	0.49	0.627
	After Spline Matching	3.7	0.72	0.472	−0.7	−0.13	0.894	−14.7	−1.32	0.187
Number of Children with High School Education or above	Before	15.6	4.20	0.000	−17.5	−4.61	0.000	−69.3	−8.24	0.000
	After One-to-Four Matching	1.8	0.35	0.724	1.2	0.25	0.802	3.0	0.46	0.644
	After Caliper Matching	−2.0	−0.38	0.701	2.6	0.56	0.573	−0.6	−0.09	0.932
	After Kernel Matching	1.6	0.31	0.755	−3.4	−0.73	0.466	−22.8	−2.84	0.005
	After Spline Matching	−1.5	−0.28	0.777	3.2	0.69	0.491	−2.1	−0.31	0.755
Co-Living	Before	23.2	6.32	0.000	14.9	4.16	0.000	−3.0	−0.44	0.657
	After One-to-Four Matching	−2.1	−0.41	0.682	−5.2	−1.03	0.303	−5.6	−0.58	0.559
	After Caliper Matching	−2.5	−0.48	0.634	−1.2	−0.25	0.804	0.6	0.06	0.953
	After Kernel Matching	2.6	0.49	0.622	4.0	0.80	0.424	−1.2	−0.12	0.902
	After Spline Matching	−1.3	−0.25	0.799	−4.5	−0.90	0.371	−6.8	−0.71	0.478
Nearby Living	Before	−14.9	−3.78	0.000	0.7	0.19	0.850	45.3	7.27	0.000
	After One-to-Four Matching	3.0	0.65	0.518	0.2	0.04	0.972	−5.3	−0.51	0.610
	After Caliper Matching	1.9	0.41	0.680	0.0	0.00	0.998	−0.2	−0.02	0.984
	After Kernel Matching	−0.8	−0.17	0.865	0.5	0.10	0.917	9.9	0.97	0.334
	After Spline Matching	2.0	0.42	0.678	−6.9	−1.37	0.169	1.0	0.10	0.924
Financial Support	Before	−20.1	−5.68	0.000	−1.2	−0.33	0.745	26.0	3.37	0.001
	After One-to-Four Matching	−0.6	−0.11	0.909	−1.1	−0.23	0.820	−3.4	−0.45	0.654
	After Caliper Matching	0.6	0.11	0.910	−0.3	−0.07	0.947	−0.0	−0.00	0.997
	After Kernel Matching	−3.3	−0.61	0.540	−0.2	−0.05	0.961	7.8	0.92	0.358
	After Spline Matching	4.1	0.75	0.451	1.3	0.26	0.796	−1.4	−0.18	0.861

**Table A3:** The estimation results of logit model for children’s marital statuses.

Variables	Single		Divorced/Separated		Widowed	
	b	SE	b	SE	b	SE
Age	−0.069 ***	0.007	0.001	0.006	0.092 ***	0.012
Gender	0.134	0.080	−0.024	0.078	−0.223	0.151
Education	0.217	0.117	0.133	0.126	−1.003	0.526
Marital Status (ref., married)						
Widowed	0.124	0.122	0.101	0.107	0.002	0.178
Divorced/Separated	0.407	0.295	0.574 *	0.274	0.209	0.659
Disability	0.068	0.084	−0.157	0.080	−0.213	0.152
Number of Chronic Diseases	−0.035	0.022	0.074 ***	0.019	−0.014	0.038
Monthly Household Consumption	−0.175 ***	0.042	−0.016	0.041	−0.033	0.072
Region	−0.009	0.089	0.277 ***	0.084	−0.048	0.166
Number of Living Children	0.360 ***	0.034	0.173 ***	0.032	0.345 ***	0.047
Number of Deceased Children	0.085	0.058	−0.049	0.059	0.083	0.080
Proportion of Children with Poor Health	0.808 ***	0.213	0.894 ***	0.175	0.313	0.347
Proportion of Children with High School Education or above	0.674 ***	0.111	−0.375 ***	0.113	−1.255 ***	0.312
Co-Living	0.445 ***	0.082	0.289 ***	0.080	−0.034	0.163
Nearby Living	−0.288 **	0.109	−0.174	0.093	0.210	0.151
Financial Support	−0.478 ***	0.099	−0.104	0.105	0.018	0.265
Constant	2.452 ***	0.491	−2.737 ***	0.466	−10.414 ***	0.944
Log pseudolikelihood	−2404.459		−2604.668		−808.749	
Pseudo R^2^	0.055		0.027		0.186	
N	7838		7896		7278	

Note: * *p* < 0.05, ** *p* < 0.01, *** *p* < 0.001.
